# Visualization of Altered Hippocampal Connectivity in an Animal Model of Alzheimer’s Disease

**DOI:** 10.1007/s12035-018-0918-y

**Published:** 2018-02-27

**Authors:** Seong Gak Jeon, Yong Jun Kim, Kyoung Ah Kim, Inhee Mook-Jung, Minho Moon

**Affiliations:** 1 Department of Biochemistry, College of Medicine, Konyang University, Daejeon, 302-718 Republic of Korea; 2 Department of Pathology, College of Medicine, Kyung Hee University, Seoul, 02447 Republic of Korea; 3 Department of Biochemistry and Biomedical Sciences, Seoul National University College of Medicine, Seoul, 03080 Republic of Korea

**Keywords:** Alzheimer’s disease, Hippocampus, 5XFAD mice, Retrograde tracer, DiI

## Abstract

Alzheimer’s disease (AD) is a neurodegenerative disorder characterized by cognitive decline and neurodegeneration in the hippocampus. Despite the pathological importance of the hippocampal degeneration in AD, little topographical evidence exists of impaired hippocampal connectivity in patients with AD. To investigate the anatomical connections of the hippocampus, we injected the neurotracer 1,1′-dioctadecyl-3,3,3′3,3′-tetramethyl-indocarbocyanine perchlorate (DiI) into the hippocampi of 5XFAD mice, which were used as an animal model of AD. In wild-type controls, DiI-containing cells were found in the entorhinal cortex, medial septum, locus coeruleus, dorsal raphe, substantia nigra pars compacta, and olfactory bulb. Hippocampal inputs were decreased in multiple brain regions in the 5XFAD mice compared to wild-type littermate mice. These results are the first to reveal alterations at the cellular level in hippocampal connectivity in the brains of 5XFAD mice. These results suggest that anatomical mapping of hippocampal connectivity will elucidate new pathogenic mechanisms and therapeutic targets for AD treatment.

**Electronic supplementary material:**

The online version of this article (10.1007/s12035-018-0918-y) contains supplementary material, which is available to authorized users.

## Introduction

Alzheimer’s disease (AD), which is the most divesting age-dependent neurodegenerative disorder, is characterized by memory and cognition impairments. The severity of the pathology in patients with AD correlates with their burden of plaques formed by amyloid-beta (Aβ) protein and neurofibrillary tangles resulting from hyperphosphorylation of the microtubule-associated protein tau [1]. Aβ accumulation in the brain is a major causative factor of AD pathogenesis, and it results in neuroinflammation, neuronal loss, and synaptic dysfunction. These histological pathologies are associated with cognitive decline and memory impairment [2]. Among them, the cognitive dysfunction of patients with AD is primarily caused by synaptic loss, which is indicated by decreased levels of synaptic proteins, such as synaptophysin and postsynaptic density protein 95, in the brains of patients with AD [1, 3].

The hippocampus is involved in spatial and episodic memory in humans [4]. In addition, patients with damaged hippocampi show impaired declarative and semantic memory [5, 6]. Hippocampal degeneration, which is the most obvious feature of patients with AD, results in symptoms of deteriorating cognitive functions, olfactory impairments, and emotional deficits [7–9]. Especially, the entorhinal cortex (EC) is not the only major brain region that sends axons to the hippocampus, but it is the first brain area that is affected by AD pathogenesis [10]. The neuronal pathways from the EC to the hippocampus are indispensable for cognitive functions, including memory retrieval and initial memory acquisition [11]. Interestingly, the olfacto-hippocampal network has a critical role in odor-discrimination learning [12], and a correlation has been reported between olfactory deficits and cognitive function in AD patients [13–16]. The hippocampus innervates many brain regions involved in cognition. In addition to the glutamatergic inputs of the hippocampus, it receives dopaminergic, noradrenergic, serotonergic, and cholinergic inputs [17–20]. The hippocampus receives dopaminergic inputs from the substantia nigra (SN) [21], and these inputs are associated with cognitive function and adult hippocampal neurogenesis [22, 23]. Moreover, the pathogenesis of AD is, in part, associated with dopaminergic neuronal loss and deficits [24]. The locus coeruleus (LC) provides noradrenergic inputs and is a major source of noradrenaline to the hippocampus, which has a critical role in cognitive functions [25]. Interestingly, neuronal degeneration in the LC is a well-known early pathology of AD [26, 27]. The dorsal raphe (DR), which is the largest serotonergic nucleus, directly innervates the hippocampus [28]. Furthermore, the DR is strongly associated with neuropsychiatric symptoms, such as agitation, depression, and anxiety, which are observed in patients with AD [29]. The major cholinergic projections to the hippocampus originate from the medial septum (MS) [30]. Decreased cholinergic innervation of the hippocampus has been demonstrated to impair learning and memory in rodents and monkey [31–33]. In addition, cholinergic dysfunction is one of the major abnormalities in patients with AD [34, 35]. To date, the loss of hippocampal inputs from extrahippocampal areas has been indirectly demonstrated in histological and electrophysiological studies. Moreover, functional neuroimaging studies have been conducted to identify the exact hippocampal connections that underlie the symptoms of AD [36, 37]. However, no previous anatomical studies have reported evidence of the topographical destruction of hippocampal pathways or the level of impairment in hippocampal inputs in the brains with AD.

One of the main purposes of neuroscience research is the investigation of the integration of neuronal assemblies in the brain into neural circuits that control behavior. Therefore, the visualization of specific neural pathways is critical for understanding the relationship between structure and function in the central nervous system. Recently, a number of studies extensively mapped neuronal connectivity in the brains of animals and humans. Neuronal connectivity in animal brains can be examined at the microscale, mesoscale, and macroscale level [38]. At the mesoscale level, various neuroanatomical tracers are used to visualize brain connectivity. Classically, neural circuit systems have been delineated with tracers that reveal the projections of subsets of neurons. Tract-tracing with neurotracers is unavoidable in studies of neuronal circuitry and its related neuronal functions under healthy and disease conditions. However, to date, few studies of mesoscale brain connectivity have been conducted with neuroanatomical tracing in the brains of patients with AD.

Cognitive dysfunction in patients with AD is mainly caused by synaptic degeneration in the hippocampus, which has been demonstrated by the expression of presynaptic terminal proteins, such as synaptophysin [1, 3]. Unfortunately, because presynaptic markers only label presynaptic axon terminals [39], they do not provide information on which brain region is the origin of the presynaptic axon terminals. Various neuronal tracers, such as engineered viruses, tracer proteins, and dyes, can be used to visualize neuronal or synaptic connections and map complex neuronal connections in the central nervous system [40]. Neural tracers, which are injected into or applied to the brain, can be taken up by endocytosis into neurons and transported in axons and dendrites in both anterograde and retrograde directions, where they can be visualized by immunohistochemical techniques. Trans-synaptic tracers can be transported from one neuron to another at or near synapses, thus revealing the locations of connected neurons [41].

To examine whether hippocampal connectivity is changed by AD-related pathologic proteins, we aimed to elucidate hippocampal afferents in an animal model of AD with 1,1′-dioctadecyl-3,3,3′3,3′-tetramethyl-indocarbocyanine perchlorate (DiI), which is a retrograde neurotracer that has been widely used in cells and tissues because it does not affect cell viability, development, or basic physiological properties [42–45]. When the neurotracer DiI is injected into the brain, it is taken up by axon terminals and retrogradely transported to the cell body, thereby tracing various afferent inputs from multiple brain regions. By using the DiI tract-tracing technique, we were able to visualize retrogradely labeled inputs of the hippocampus from several brain regions and quantify impaired hippocampal connections in the AD animal model. The analyses of the DiI-positive neurons in the brains of the 5XFAD mice provided direct evidence for topographical changes in hippocampal connectivity and impaired hippocampal afferents in AD.

## Materials and Methods

### Animals

Transgenic mice with five familial mutations of AD-related genes (5XFAD mice) express three mutations in human APP (K670N/M671L, V717I, and I716V) and two mutations in human PSEN1 (M146L and L286V) [46]. The animals were obtained from The Jackson Laboratory (Bar Harbor, ME, USA; catalog number 006554). The wild-type littermates and 5XFAD mice were genotyped and used in the present study. The maintenance and treatment of the animals were performed in accordance with the Guide for the Care and Use of Laboratory Animals (NIH Publication No. 85-23, revised 1985) and the Animal Care and Use Guidelines of Konyang University (Daejeon, Korea).

### Stereotaxic Neurotracer Injections

For the topographical tracing, DiI (Sigma-Aldrich Corporation, St. Louis, MO, USA) or Fluoro-Gold (Fluorochrome, LLC, Denver, CO, USA) was dissolved in dimethyl sulfoxide to result in a 20-mM stock solution. The stock solution was diluted to 82 μM with phosphate-buffered saline (PBS) and then used as the injection solution. When the mice were 11.5 months old, the DiI or Fluoro-Gold were stereotaxically injected at 1 μL/min for 3 min into the dentate gyrus of the hippocampus (AP, − 2.0 mm; ML, 1.3 mm; DV, − 1.9 mm from bregma and skull) in the 5XFAD (*n* = 5) and wild-type mice (*n* = 5), while they were anesthetized with Avertin (tribromoethanol; Sigma-Aldrich Corporation, St. Louis, MO, USA; 250 μg/kg), which was administered by intraperitoneal injections. After the Dil or Fluoro-Gold injections, the needle was slowly withdrawn and the skin was sutured.

### Brain Tissue Preparation

Four days after the injections, the animals were anesthetized, transcardially perfused with 0.05 M PBS, and then fixed with ice-cooled 4% paraformaldehyde in 0.1 M phosphate buffer (PB). The brain tissue was extracted, postfixed in 0.1 M PB containing 4% paraformaldehyde for 20 h at 4 °C, and then saturated with a 30% sucrose in 0.05 M PBS solution for 3 days at 4 °C for cryoprotection. The samples were embedded with optimal cutting temperature (OCT) compound and cut into serial 30-μm-thick coronal sections with a cryostat (Leica Biosystems, Wetzlar, Germany). The tissue sections were stored in a cryoprotectant (25% ethylene glycol, 25% glycerol, and 0.05 M PB) at 4 °C until they were needed for histology.

### Image Acquisition and Analysis

To trace the DiI-labeled cells, the entire tissue section was imaged with a Zeiss LSM 700 Meta confocal microscope (Carl Zeiss AG, Oberkochen, Germany; *λ*_ex_ = 550 nm; *λ*_em_ = 567 nm). The DiI-labeled cell bodies were examined in several brain regions, including the olfactory bulb (OB), MS, EC, substantia nigra pars compacta (SNc), interfascicular region of the DR nucleus, and locus coeruleus (LC). To quantify the DiI-labeled cells, 4–10 representative images of each region were analyzed and quantified by ImageJ software (National Institutes of Health (NIH), Bethesda, MD, USA) as following steps: (1) images are converted to 8 bit for the quantify images; (2) following converting, images are thresholded for area of DiI-positive cells and background signals are removed; (3) topographic anatomical areas are designated based on DAPI counterstaining; (4) thresholded images for the designated brain area are quantified by Analyze particles tool to the “% Area” value of the DiI-positive cells; (5) to normalize relative to the control, the following equations are applied to the “% Area” values of the two groups (WT and 5XFAD) to be compared: %of control = (%Area_WT or 5XFAD_/ % Area_average of WT_) × 100.

### Statistical Analyses

The data are presented as the mean ± standard error of the mean. The differences between the two groups were analyzed statistically with independent *t* tests and Prism 5 (Windows Version 3.10; Systat Software, Inc., San Jose, CA, USA). *P* value less than 0.05 indicated statistical significance.

## Results

### Histological Profiling of the Neuronal Inputs of the Hippocampus in Healthy Mice

**Fig. 1:**
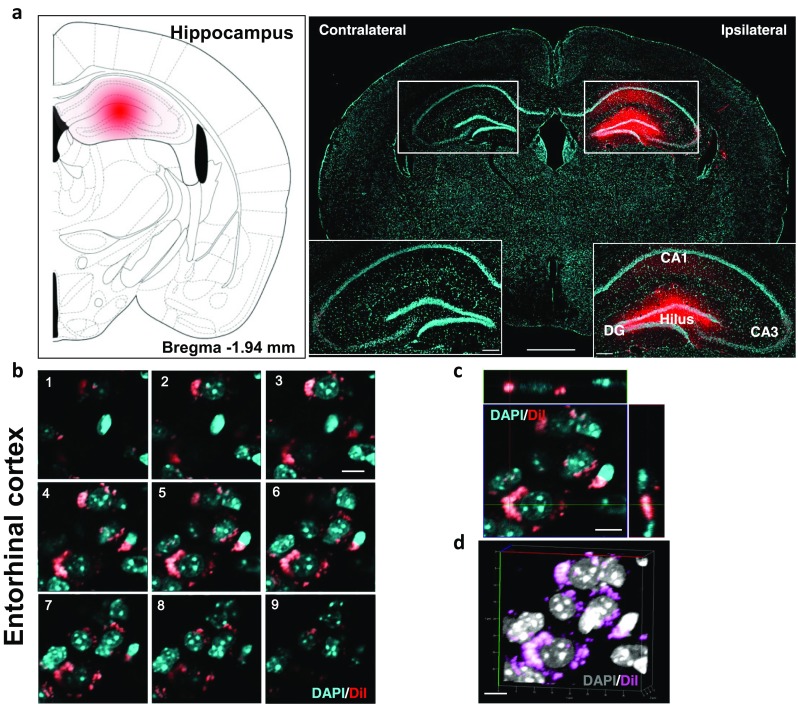
Validation and characterization of the application of the DiI neurotracer in the brains of wild-type mice. **a** Photomicrographic validation of the stereotaxic injection sites of DiI. The mouse brain atlas diagram illustrates the injection sites. Scale bars = 1 mm and 200 μm for the magnified inserts. **b** Serial Z-stack images, comprising nine sections, of the subcellular localization of DiI that was retrogradely transported from the hippocampus to the entorhinal cortex. **c** Orthogonal view of the z-stack images shown in **b**. The panels on the side and bottom show *y*–*z* and *x*–*z* cross-sectional images, respectively. **d** The three-dimensional Z-projection of the acquired stacks. DAPI was used to stain the nuclei. Scale bars = 10 μm **b**–**d**

**Fig. 2:**
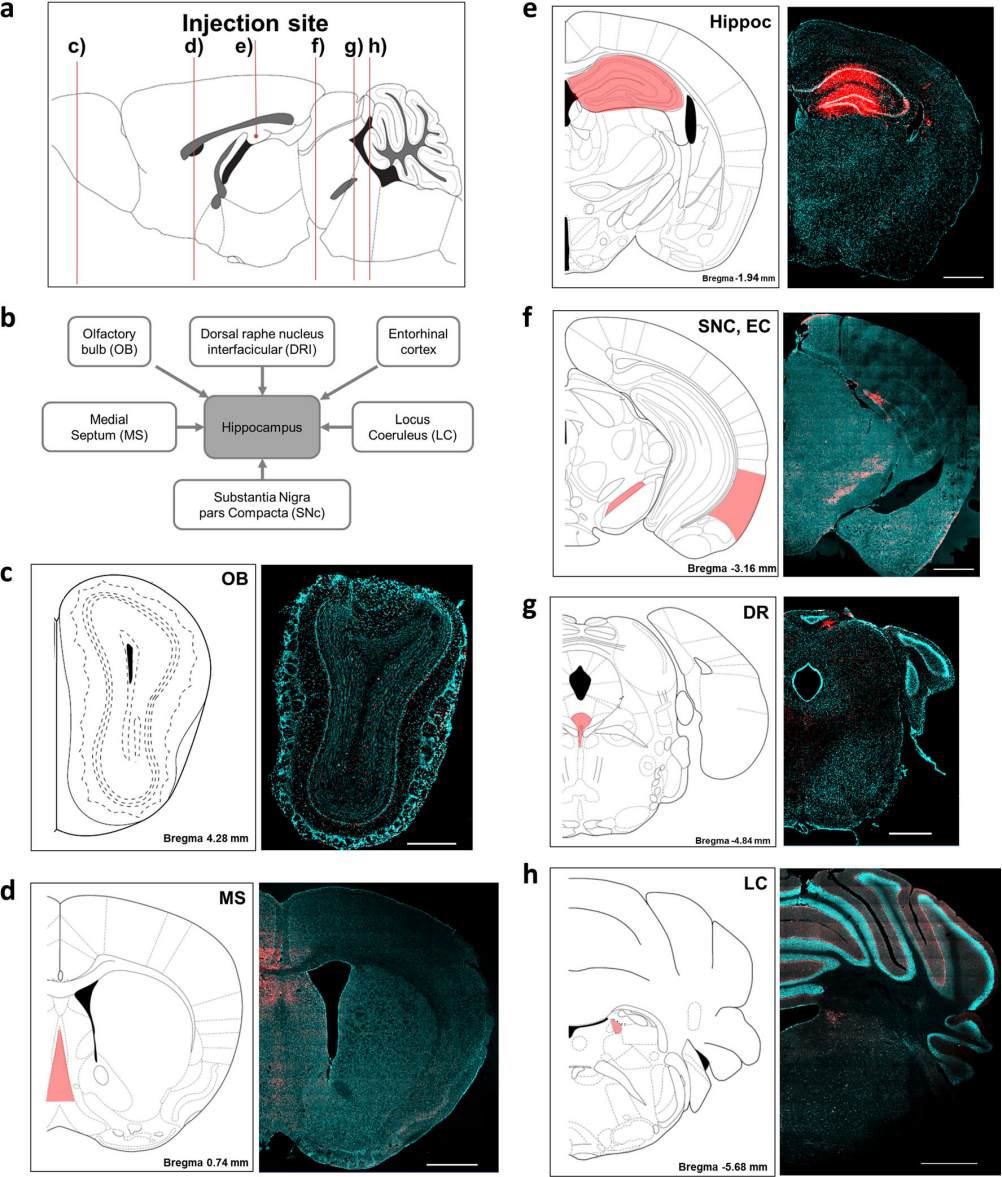
Delineating the neural inputs of the hippocampus in the wild-type mice with the neurotracer DiI. **a** A sagittal view of mouse brain showing representative figures of (c–h). **b** Schematic drawing of the brain regions that project axons to the hippocampus. DiI fluorescence was observed in **c** the olfactory bulb (OB), **d** medial septum (MS), **e** hippocampus (Hippoc), **f** substantia nigra pars compacta (SNc), entorhinal cortex (EC), **g** dorsal raphe (DR), and **h** locus coeruleus (LC). All tissues were counterstained with DAPI (blue). All figures were captured in the ipsilateral hemisphere. Scale bars = 1 and 500 μm for **c**

To determine the origin of axonal inputs of the hippocampus, we used DiI, a retrograde tracer, to identify extrahippocampal afferents. Before the afferent inputs of the hippocampus were examined in the AD model brains, we injected the retrograde tracer into the hili of the hippocampi of wild-type littermate mice to validate the injection site and delineate the afferents of the hippocampus. Four days after the injection, DiI fluorescence was detected in the dentate gyrus, CA1, and CA3 (Fig. 1a). DiI signals were also found in the contralateral dentate gyrus and CA3, and these signals represent projections from the contralateral hippocampus (Supplementary Fig. 1). Because the hippocampus receives major inputs from the EC [47, 48], we performed Z-stack imaging of the (DAPI) and DiI signals in the EC of the wild-type mice to investigate the subcellular localization of the DiI that was axonally transported from the hippocampus. The fluorescence signals of the retrotransported DiI were localized in the cytoplasm of the DAPI-stained cells (Fig. 1b–d). Fluorescent cells were prominent in specific brain regions or nuclei, including the MS, LC, DR, SNc, and OB, in the DiI-injected littermate controls (Fig. 2a–h). These labeled regions are known to send afferents to the hippocampus, and these results indicated that the DiI-positive cells were afferent neurons of the hippocampus. In addition, the number of DiI-containing cells in the injected hemisphere was larger than that of the contralateral side, which suggested that the hippocampus receives neural inputs from extrahippocampal areas in a predominantly ipsilateral manner (Supplementary Fig. 2). Although DiI is anterogradely and retrogradely transported in neurons, the properties of the anterograde transport have not yet been clarified [43, 49]. To confirm the retrograde transport of DiI, we traced another retrograde tracer, Fluoro-Gold [50]. As a result, we found intrahippocampally injected Fluoro-Gold in the same extrahippocampal regions observed in the DiI-injected brains (Supplementary Fig. 3).

### Altered Olfacto-Hippocampal Pathways in the 5XFAD Mice

**Fig. 3:**
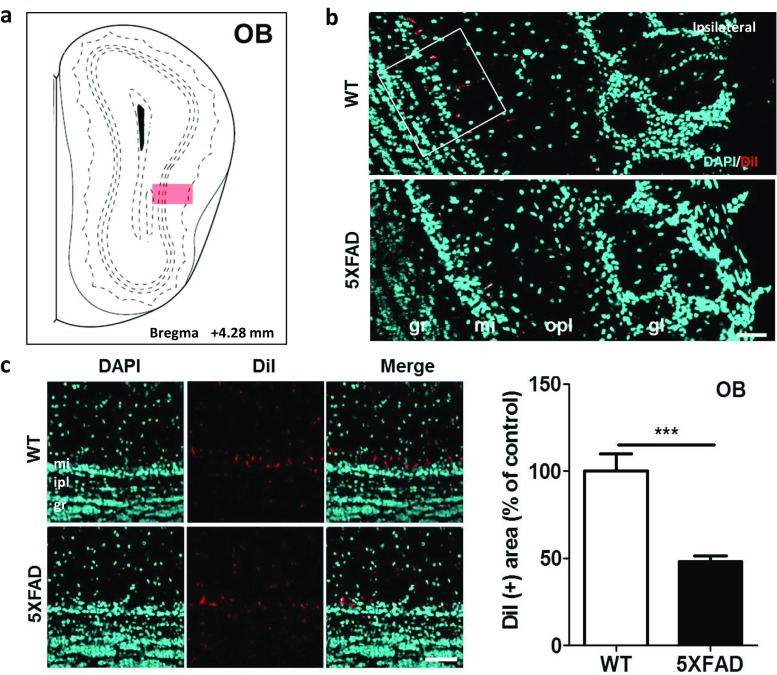
Olfactory input to the hippocampus was significantly decreased in the 5XFAD mice compared to the wild-type littermate mice. **a** Diagram of the coronal mouse brain sections illustrating the location of the OB at bregma + 4.28 mm. **b** Representative figures showing DiI-positive somata in the main OB (MOB). DiI-positive signals are mainly observed in the mitral layer of the OB. **c** Quantification of the DiI-positive area in the MOB. Scale bar = 50 μm. The values are given as mean ± standard error of the mean. ^***^p < 0.001 indicates significant differences between the groups. OB, olfactory bulb; gr, granule layer; ipl, inner plexiform layer; mi, mitral layer; opl, outer plexiform layer; gl, glomerular layer

To investigate the neural connections between the OB and the hippocampus in the AD model brains, DiI was injected into the hippocampi of the wild-type and 5XFAD mice when they were 11.5 months old. Four days after the DiI injections, DiI fluorescence was observed in the mitral layer of the OB in both the 5XFAD and wild-type mice (Fig. 3a, b). The number of DiI-positive cells was decreased in the OB of the 5XFAD mice compared with those in the wild-type mice (Fig. 3c, *t* = 4.966, *p* = 0.0006). These results suggested that the olfactory memory deficits in patients with AD are associated with decreased innervation of the hippocampus from the OB.

### Disrupted Septohippocampal Projections in the 5XFAD Mice

**Fig. 4:**
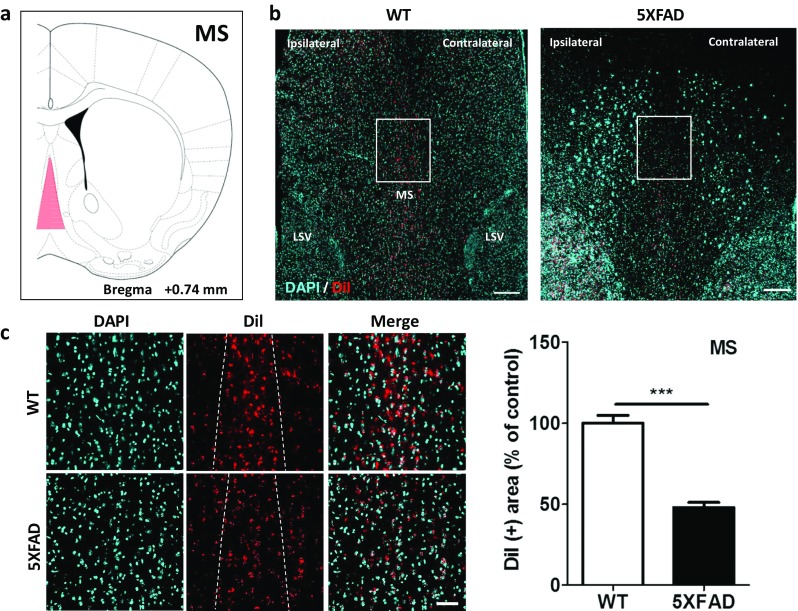
Medial septal inputs to the hippocampus were significantly decreased in the 5XFAD mice compared to their littermate controls. **a** Diagram of a mouse brain atlas illustrating the location of the MS at Bregma + 0.74 mm. **b** Representative figures of DiI-containing somata in the MS complex. DiI-positive cells are mainly observed in the MS nucleus. Scale bar = 200 μm. **c** Magnification of the white rectangles in **b**. **d** Quantification of the DiI-positive area in the MS nucleus. Scale bar = 50 μm. ^***^p < 0.001 indicates significant differences between the groups. MS, medial septal nucleus; LSV, lateral septal nucleus, ventral part

To examine if the septohippocampal pathways were altered in Aβ-overexpressing brains, we traced the transport of the retrograde tracer from the hippocampi of the 5XFAD mice. Fluorescence was observed in septal areas in both 5XFAD and wild-type mice (Fig. 4a, b). Compared to the wild-type mice, the DiI-labeled afferents from the MS were significantly decreased by 52% in the 5XFAD mice (Fig. 4c, *t* = 9.132, *p* < 0.0001). These results indicated that the innervation of the hippocampus from the MS was decreased in Aβ-overexpressing brains.

### Decreased Entorhinal-Hippocampal Connections in the 5XFAD Mice

**Fig. 5:**
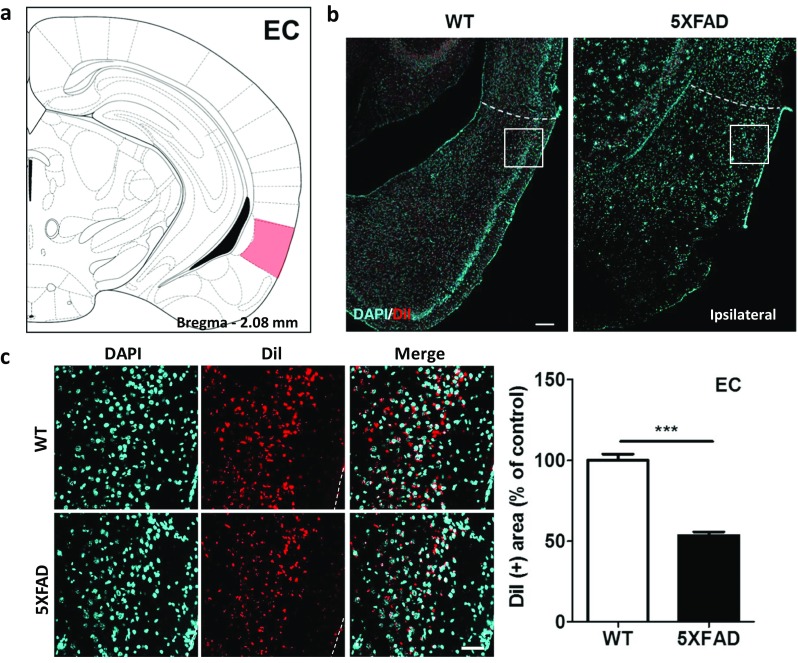
Innervation of the hippocampus from the EC was significantly impaired in the 5XFAD mice compared to the wild-type mice. **a** Diagram of a mouse brain atlas illustrating the location of the EC at bregma − 2.08 mm. **b** Representative figures of DiI-positive somata in the entorhinal area, lateral part. The DiI-positive cells are mainly observed in layer 2/3 of the EC. Scale bar = 200 μm. **c** Quantification of the DiI-positive area in the EC. Scale bar = 50 μm. ^***^p < 0.001 indicates significant differences between the groups. EC, entorhinal cortex; MS, medial septal nucleus; LSV, lateral septal nucleus, ventral part

To investigate changes in the hippocampal inputs originating from the EC, we revealed the entorhinal-hippocampal connections with the DiI tract-tracing technique. Most of the traced afferents were found in the superficial layers of the LC (Fig. 5a, b). These retrograde-tracing results demonstrated that the DiI-positive area was reduced by 46.5% in the cortical layers of the EC in the 5XFAD mice compared to that in the wild-type mice (Fig. 5c, *t* = 10.73, *p* < 0.0001). These findings suggested that the neural inputs of the hippocampus from the EC are decreased in the brains of patients with AD.

### Altered Nigrohippocampal Pathways in the 5XFAD Mice

**Fig. 6:**
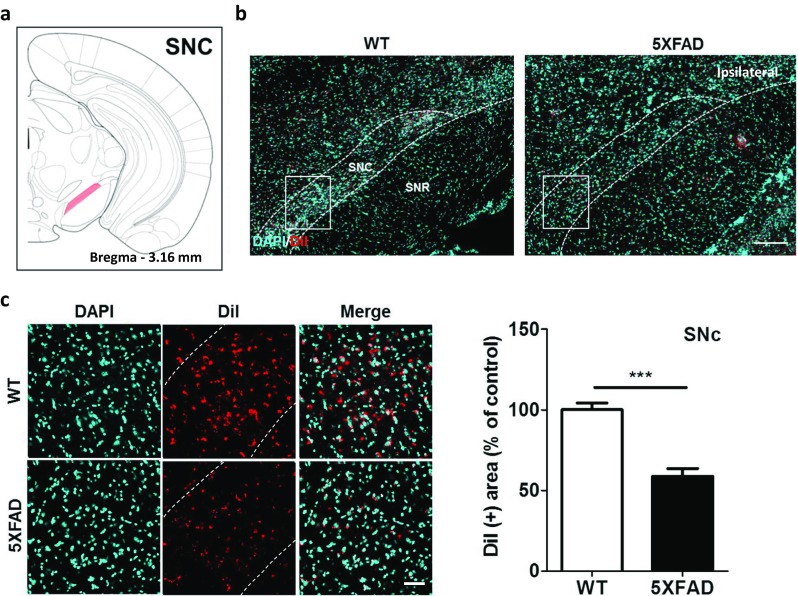
The nigrohippocampal pathways were impaired in the 5XFAD mice compared to the wild-type mice. **a** Diagram of a mouse brain atlas illustrating the location of the SNc at bregma − 3.16 mm. **b** Representative figures of DiI-positive somata in the entorhinal area, lateral part. DiI-positive cells were mainly observed in the SNc. Scale bar = 200 μm. **c** Quantification of the DiI-positive area in the SNc. Scale bar = 50 μm. ^***^p < 0.001 indicates significant differences between the groups. SNc, substantia nigra, compact part; SNR, substantia nigra, reticular part

To reveal the nigrohippocampal pathway, we injected DiI into the hippocampus and examined the midbrain (Fig. 6a, b). Compared with the littermate mice, the nigrohippocampal pathway was decreased by 41.3% in the SNc of the 5XFAD mice, which indicated that this pathway was significantly altered in the 5XFAD mice (Fig. 6c, *t* = 6.496, *p* < 0.0001). These retrograde tract-tracing results showed that hippocampal afferents from the SNc were significantly decreased in Aβ-overexpressing brains.

### Disrupted Raphe-Hippocampal Projections in the 5XFAD Mice

**Fig. 7:**
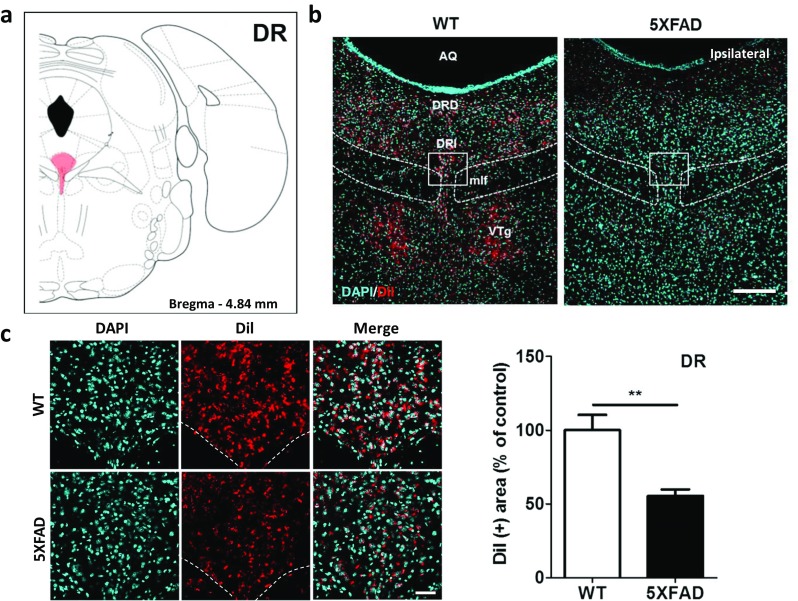
The innervation of the hippocampus from the DR was significantly decreased in the 5XFAD mice compared to the wild-type mice. **a** Diagram of a mouse brain atlas illustrating the location of the DR at bregma − 4.84 mm. **b** Representative figures of DiI-positive somata in the midbrain raphe nuclei. DiI-positive cells were mainly observed in the DR and VTg. Scale bar = 200 μm. **c** Quantification of the DiI-positive area in the DRI. Scale bar = 50 μm. ^**^p < 0.01 indicates significant differences between the groups. AQ, cerebral aqueduct; DR, dorsal raphe; DRD, dorsal raphe nucleus, dorsal part; DRI: dorsal raphe nucleus, interfascicular part; mlf, medial longitudinal fasciculus; VTg, ventral tegmental nucleus

To examine raphe-hippocampal projections, we conducted retrograde-tracing with DiI. These retrograde-tracing results demonstrated that the DiI-positive area was reduced by 44.6% in the DR of the 5XFAD mice compared with the littermate controls (Fig. 7, *t* = 3.961, *p* = 0.0027) and implied that impairments in the raphe-hippocampal projections contribute to the decreased levels of serotonin that have been observed in the hippocampi of patients with AD.

### Decreased LC-Hippocampal Innervation in the 5XFAD Mice

**Fig. 8:**
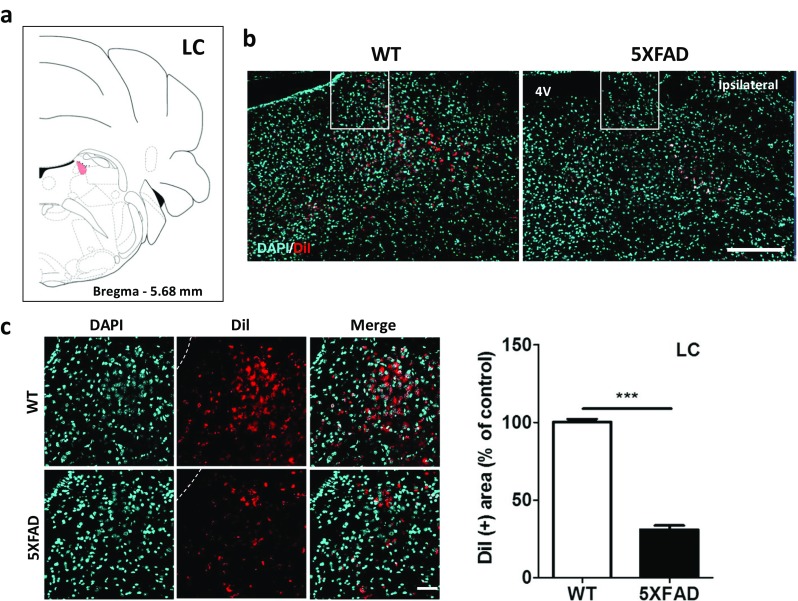
The LC-hippocampal pathways were dramatically decreased in the 5XFAD mice compared with the wild-type mice. **a** Diagram of a mouse brain atlas illustrating the location of the LC at bregma − 5.68 mm. **b** Representative figures of DiI-positive somata in the pons. DiI-positive cells were mainly observed in the LC. Scale bar = 200 um. **c** Quantification of the DiI-positive area in the LC. Scale bar = 50 μm. ^***^p < 0.001 indicates significant differences between the groups. LC, locus coeruleus; 4V, fourth ventricle

**Fig. 9:**
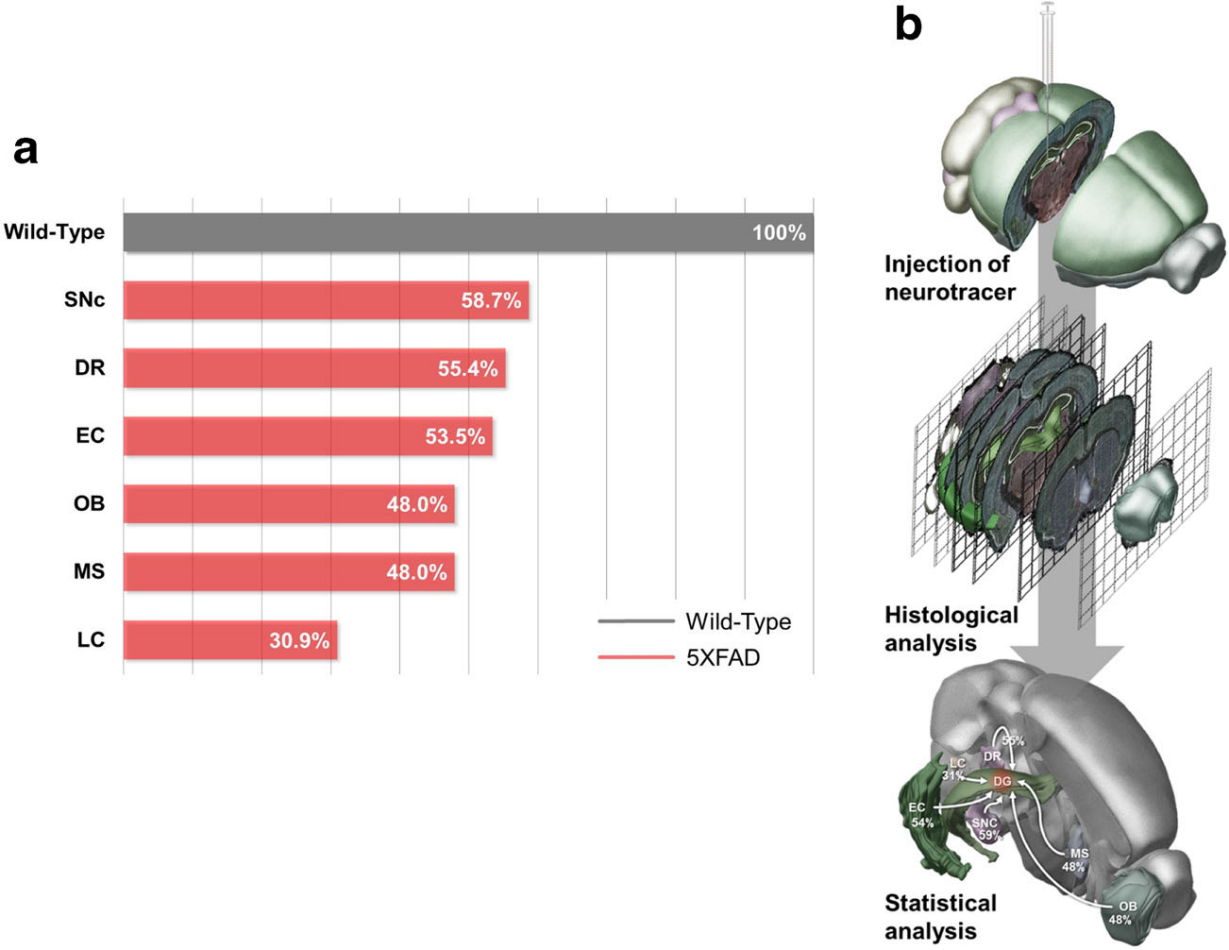
Schematic drawing of the decreased inputs into the hippocampus of 5XFAD mice. **a** A quantitative analysis confirmed that the number of DiI-positive cells was significantly decreased in the EC, MS, LC, DR, SNc, and OB of 11.5-month-old 5XFAD mice compared with wild-type littermate mice. **b** The experimental design for examining the decreased hippocampal connectivity in an animal model of AD. DiI was injected into the hippocampus of the 5XFAD mice. The tracer was taken up by axonal terminals within the hippocampus and then transported to remote regions through the axons of the neurons. Four days after the injections, histochemical analyses were conducted to show the DiI-positive hippocampal afferents

To examine alterations in hippocampal afferents from the LC in AD, we revealed LC-hippocampal projections in the 5XFAD and wild-type mice. DiI was injected into the hippocampus, and DiI fluorescence was detected in the LC of the 5XFAD and wild-type mice (Fig. 8a, b). At 11.5 months of age, the number of DiI-containing somata was dramatically decreased by 69.1% in the LC of the 5XFAD mice compared to the wild-type mice (Fig. 8c). The LC-hippocampal connection was the most severely altered of all neural pathways examined in the 5XFAD mice (Fig. 9a, *t* = 20.19, *p* < 0.0001), which supports previous reports of neuronal loss in the LC being most severe in patients with AD [51].

## Discussion

The hippocampus is the major brain region involved in the regulation of learning and memory. Due to the critical role of the hippocampus in cognitive functions, hippocampal connectivity has been examined in the brains of patients with AD. Although functional magnetic resonance imaging can be used to visualize and examine neural networks in the brains of patients with AD, it cannot be used to examine direct neuronal connections at the cellular level [36, 37]. The aim of this study was to identify topographical changes in hippocampal connectivity in AD by providing direct anatomical evidence of the origins of axon terminals that innervate neurons in the hippocampus and the extent they are damaged in patients with AD. To reveal the innervation of the hippocampus from several brain regions, we performed stereotaxic injections of the retrograde tracer DiI into the hippocampi of 5XFAD and wild-type mice. Subsequently, we analyzed the DiI-positive neurons in the extrahippocampal regions and found that afferents to the hippocampus were decreased in the 5XFAD mice (Fig. 9).

To date, many of the therapeutic drugs that are used to treat AD and that target Aβ, neuroinflammation, oxidative stress, mitochondrial dysfunction, and hyperphosphorylated tau are largely unsuccessful at restoring memory. Recently, selective abnormalities in neural circuits have been shown to be the major cause of noticeable memory loss in patients with AD [52, 53]. Thus, restoring neural networks in patients with AD might directly enhance cognitive function in these patients [53, 54].

To investigate impairments in hippocampal connectivity in AD, we used 5XFAD mice, which exhibit the major features of AD. The pathological phenotypes of 5XFAD mice are the accumulation of amyloid plaques, synaptic loss, neuronal death, impaired adult hippocampal neurogenesis, and neuroinflammation [55, 56]. In patients with AD, the accumulation of Aβ is the main contributor to the compromised synaptic networks and neural circuits [57]. Thus, we confirmed Aβ deposits in the brain regions that exhibited DiI retrogradely labeled cells in the 5XFAD mice (Supplementary Fig. 4). The brains of patients with AD also exhibit amyloid plaque accumulation in the cholinergic nuclei of the basal forebrain, LC, DR, SNc, hippocampus, and EC [58], indicating that the regions with Aβ deposition in the brains of the 5XFAD mice were very similar to those in human AD brains. In addition, the 5XFAD mice develop hippocampal degeneration and deficits in memory and cognition in an age-dependent manner [59]. The hippocampus receives glutamatergic afferents from the EC, and the levels of glutamate are reduced by 8 months of age in the 5XFAD mice [60]. In addition to the glutamatergic inputs, the hippocampus receives dopaminergic, noradrenergic, serotonergic, and cholinergic inputs, and these inputs are also decreased in the hippocampi of 5XFAD mice [60]. Despite the pathological importance of hippocampal degeneration in AD, little direct anatomical data show impairments in hippocampal inputs at the mesoscale level in animal models of AD.

Deficits in the cholinergic system are strongly associated with AD-associated memory loss [61]. Therefore, acetylcholinesterase inhibitors have been used as therapeutic agents for patients with AD [62, 63]. Neural pathways from the MS mostly terminate in the hippocampus [64, 65]. Thus, the MS is one of the major sources of released acetylcholine within the hippocampus. Our results indicated that DiI-positive cells, which trace afferent paths of the hippocampus, were significantly reduced in the MS in the animal model of AD (Fig. 4), which implied that the cholinergic septohippocampal pathway might be impaired in Aβ-overexpressing brains.

Many studies have reported that dysfunction of the noradrenergic system or LC destruction in the brains of patients with AD plays critical pathogenic roles in AD-related pathologies, such as neuroinflammation, cognitive deficits, synaptic loss, and amyloidosis [27, 66–68]. Interestingly, the LC is an especially vulnerable part of the brain in patients with AD [66]. Our results showed that DiI-traced afferents from the LC to the hippocampus were impaired the most of all the neuronal pathways examined in the brains of the 5XFAD mice (Fig. 9a), which suggests that the afferent pathway from the LC to the hippocampus is the most severely altered in AD.

Although the onset of AD is clinically diagnosed by cognitive decline, several secondary symptoms, such as depression, olfactory dysfunctions, and deficits in olfactory memory, are present in patients with AD [16, 69]. Considering the association of the DR nucleus with mood regulation, destruction of the raphe-hippocampal connection might be associated with depressive symptoms in patients with AD. In addition, the present results of the anatomical mapping of the olfacto-hippocampal connection indicate that impairments in this circuit might be the underlying mechanism of the olfactory deficits in patients with AD.

The ventral tegmental area (VTA), which is directly linked to the hippocampus, is involved in the spatial memory and activity of the hippocampus and the dopaminergic response to the novelty and encoding of hippocampal-dependent memories [70–73]. In addition to the SNc, the VTA exhibits neuronal loss in patients with AD [74]. Although DiI-positive somata were not prominent in the VTA, we observed a trend of decreased DiI-positive area in the VTA (data not shown). These impaired neuronal connections might result from the degeneration of axonal terminals within the hippocampus or somata in the regions projecting axons to the hippocampus.

In the mice receiving intrahippocampal DiI injections, fluorescence was observed in the DG, CA3, and CA1 (Figs. 1a and 2e). Cortical layer II of the EC projects mainly to CA3 and DG, and cortical layer III of the EC projects primarily to CA1 and the subiculum [47]. Consistent with the results of previous studies, our results showed that DiI-positive cells were mainly found in the superficial (II and III) layers of the EC (Fig. 5b, c). The hippocampus innervates the contralateral hippocampus [75, 76]. Consistent with the results of previous studies, DiI-positive cells were also observed in the DG and CA3 of the contralateral hippocampus (Fig. 1a and Supplementary Fig. 1). Many studies have revealed that neuronal circuitries are ipsilaterally dominant in the brain. A recent study showed that there are prevalent bilateral circuitries to corresponding ipsilateral and contralateral target regions, with the ipsilateral circuits generally being stronger than the contralateral sides [38]. Specifically, the majority of LC neurons project predominantly throughout the entire brain in an ipsilateral manner (Ader et al., 1980; Waterhouse et al., 1983; Simpson et al., 1997; Room et al., 1981). Consistent with previous reports, our results also showed that the intensity of the DiI fluorescence or the number of DiI-positive cells was increased on the ipsilateral side compared with the contralateral side in the SNc and LC of the mice brains (Supplementary Fig. 2).

These results suggested that hippocampal inputs from the cholinergic MS, noradrenergic LC, serotonergic DR, dopaminergic SNc, and glutamatergic EC were impaired in 5XFAD mice. Therefore, investigations of the destruction of neurotransmitter-specific pathways should be conducted with cell-type-specific promoters. The current findings were the first to elucidate impairments in neural pathways/tracts that originate from extrahippocampal areas in animal models of AD at the mesoscale level, and these results might be potent evidence for the pathological mechanisms underlying AD. Further studies are needed to identify the change of efferent and afferent projections within not only hippocampus but also whole brain structure in AD. Based on mapping of connectivity in the AD brain, restoring impaired neural pathways with optogenetic therapy or transcranial magnetic stimulation might be potential therapeutic strategies for treating cognitive impairments in patients with AD.

## Electronic Supplementary Material

Fig. S1 Bilateral innervation of both hemispheres of the hippocampus. The fluorescence of the DiI that was injected into the hippocampus of wild-type littermate mice is observed in both ipsilateral and contralateral hippocampi (519 KB)

(1.86 MB)

Fig. S2 Comparison of the numbers of DiI-containing cells in the two hemispheres. The red shading in the mouse brain diagram indicates the locations of the SNc **a** and LC **b** in the coronal mouse brain sections. More DiI-positive cells are seen on the ipsilateral side of the DiI injection. SNc: substantia nigra, compact part, LC: locus coeruleus (515 KB )

(1.41 MB)

Fig. S3 Validation and profiling of the Fluoro-Gold-labeled afferent connections to the hippocampus. **a** Validation of the Fluoro-Gold injection site in the hippocampus. **b** Delineating the Fluoro-Gold-positive areas in the brain regions that project afferents to the hippocampus. Representative brain slices showing DiI-labeled cells. EC: entorhinal cortex, LC: locus coeruleus, MS: medial septum, DR: Dorsal raphe (311 KB)

(906 KB)

Fig.S4 Aβ-accumulation in the brains of the 5XFAD mice. The brain sections of the 5XFAD mice were stained with the 4G8 antibody to reveal Aβ plaque accumulation in the brain areas exhibiting the DiI-labeled cells (1.24 MB)

(371 KB)
