# Supplementary figures and images for: Visualization of Altered Hippocampal Connectivity in an Animal Model of Alzheimer’s Disease
Source: Mol Neurobiol. 2018 Feb 27;55(10):7886–99. doi: 10.1007/s12035-018-0918-y (PMC6132739; doi:10.1007/s12035-018-0918-y)

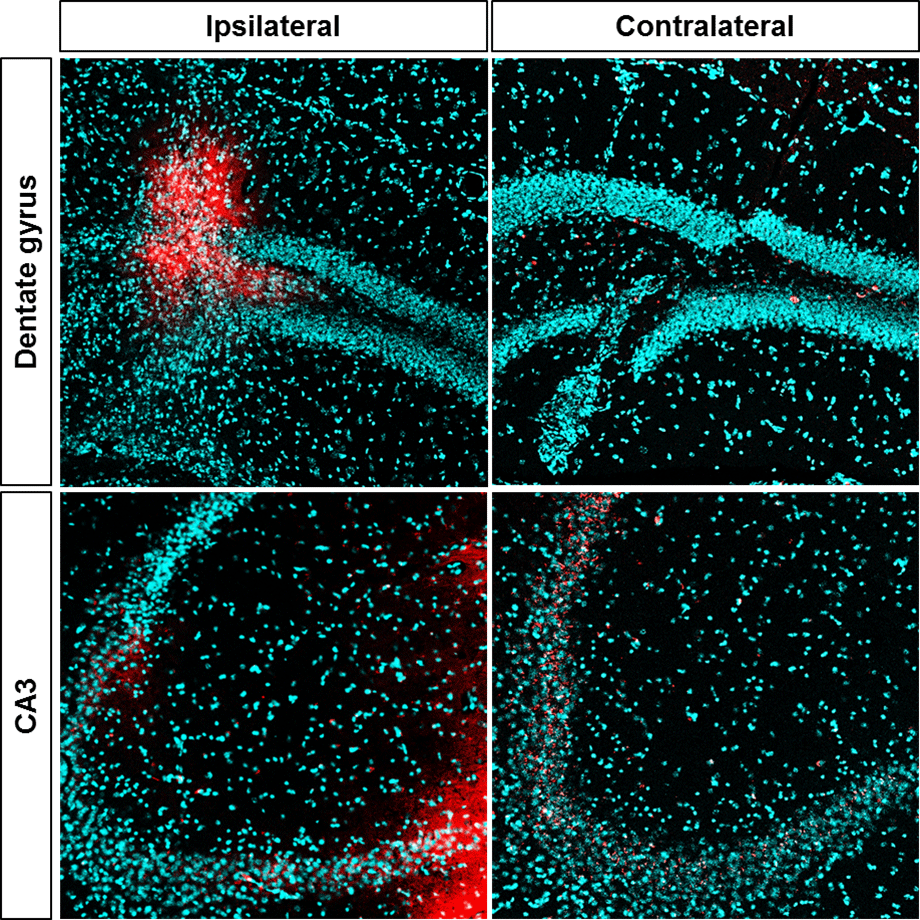

Supplement: Supplementary file 1 — Bilateral innervation of both hemispheres of the hippocampus. The fluorescence of the DiI that was injected into the hippocampus of wild-type littermate mice is observed in both ipsilateral and contralateral hippocampi (519 KB) [file 12035_2018_918_Fig10_ESM.gif]

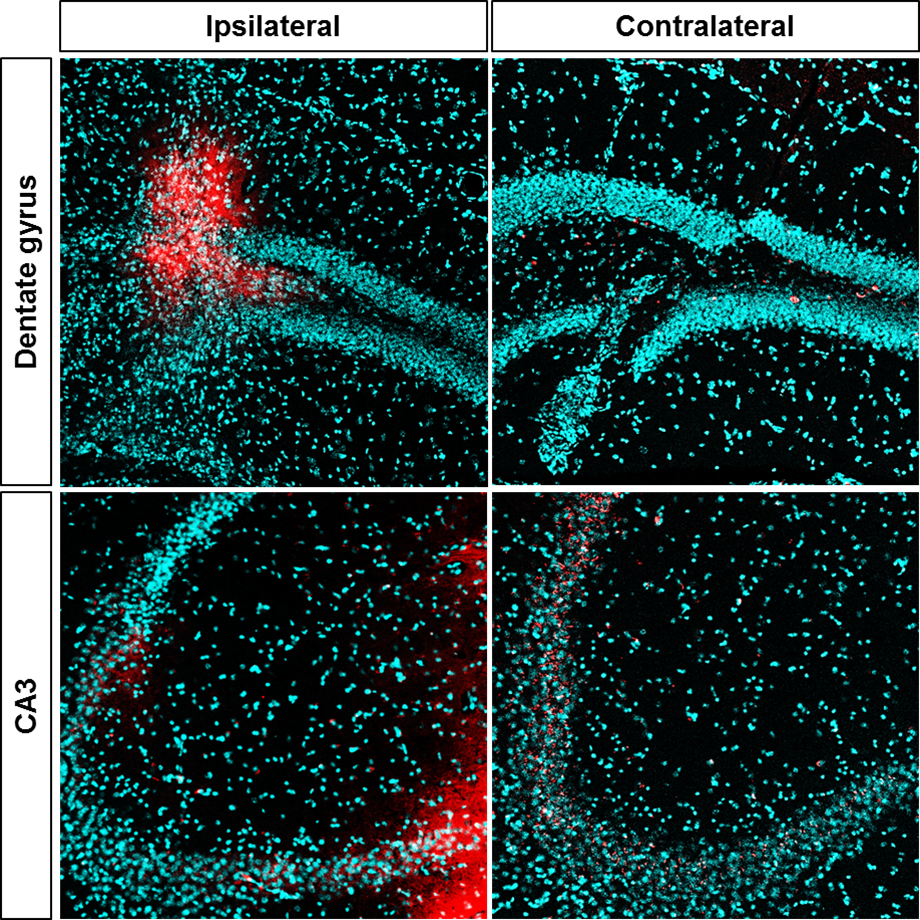

Supplement: Supplementary file 2 — (1.86 MB) [file 12035_2018_918_MOESM1_ESM.tif]

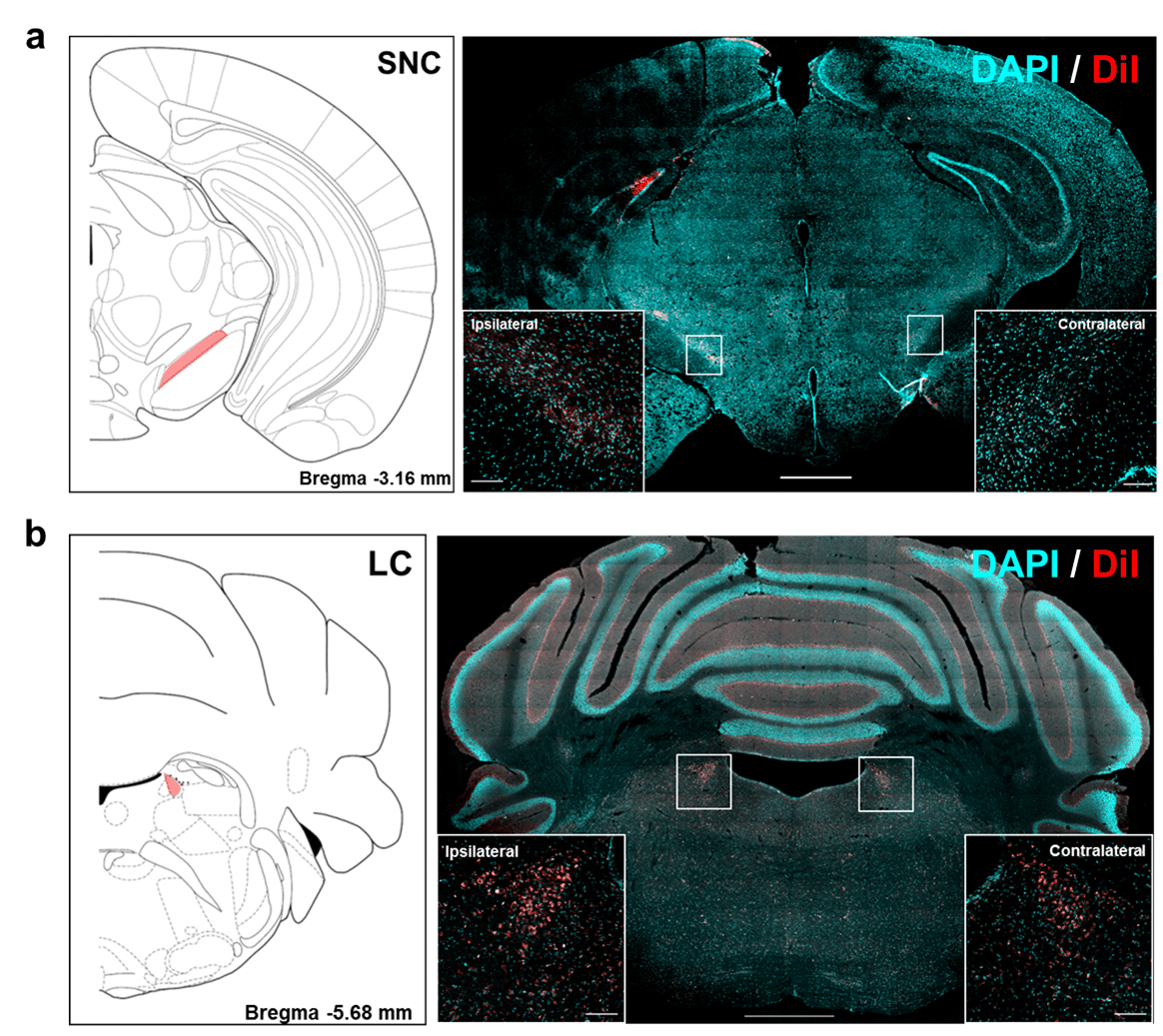

Supplement: Supplementary file 3 — Comparison of the numbers of DiI-containing cells in the two hemispheres. The red shading in the mouse brain diagram indicates the locations of the SNc a and LC b in the coronal mouse brain sections. More DiI-positive cells are seen on the ipsilateral side of the DiI injection. SNc: substantia nigra, compact part, LC: locus coeruleus (515 KB ) [file 12035_2018_918_Fig11_ESM.gif]

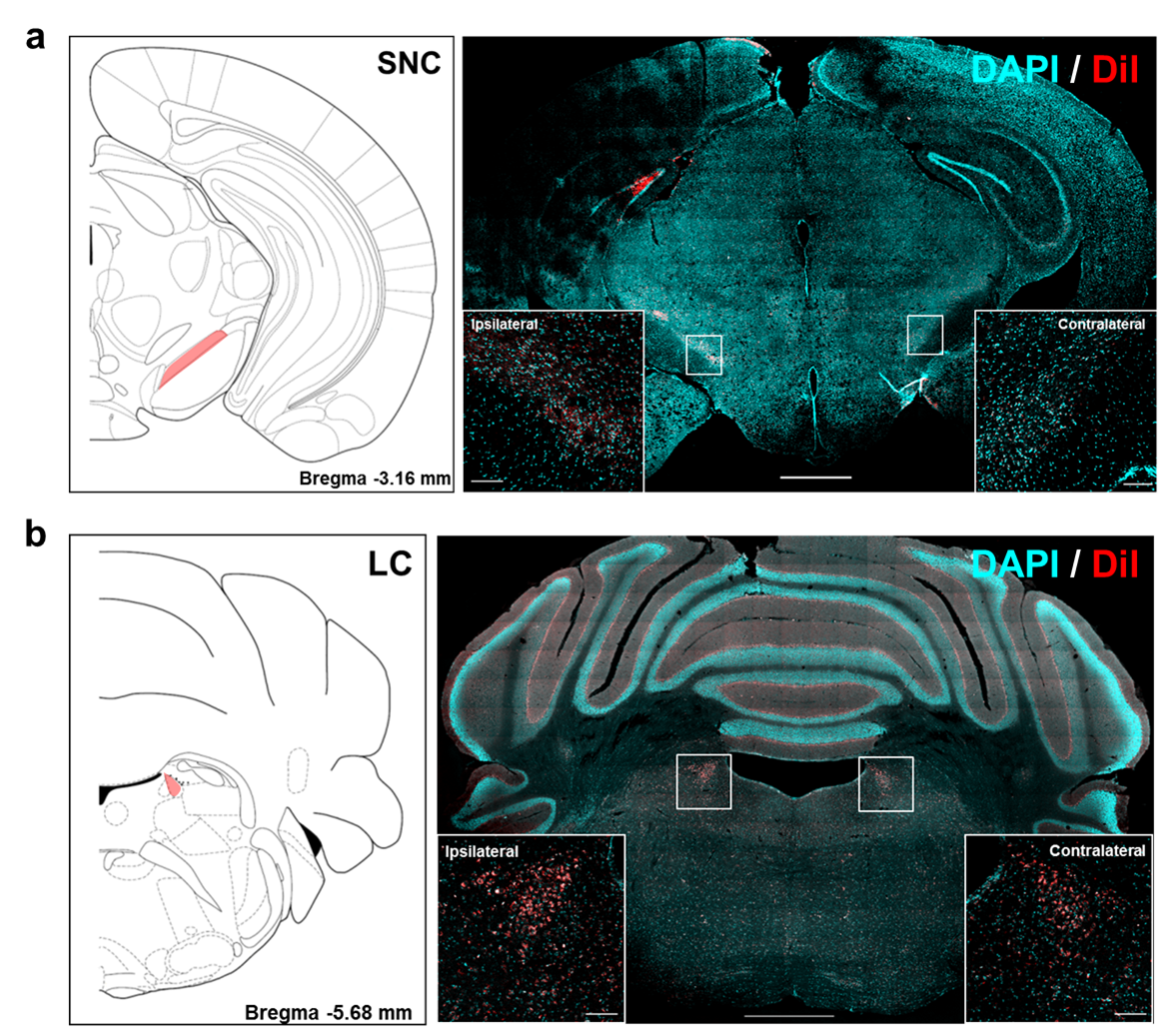

Supplement: Supplementary file 4 — (1.41 MB) [file 12035_2018_918_MOESM2_ESM.tif]

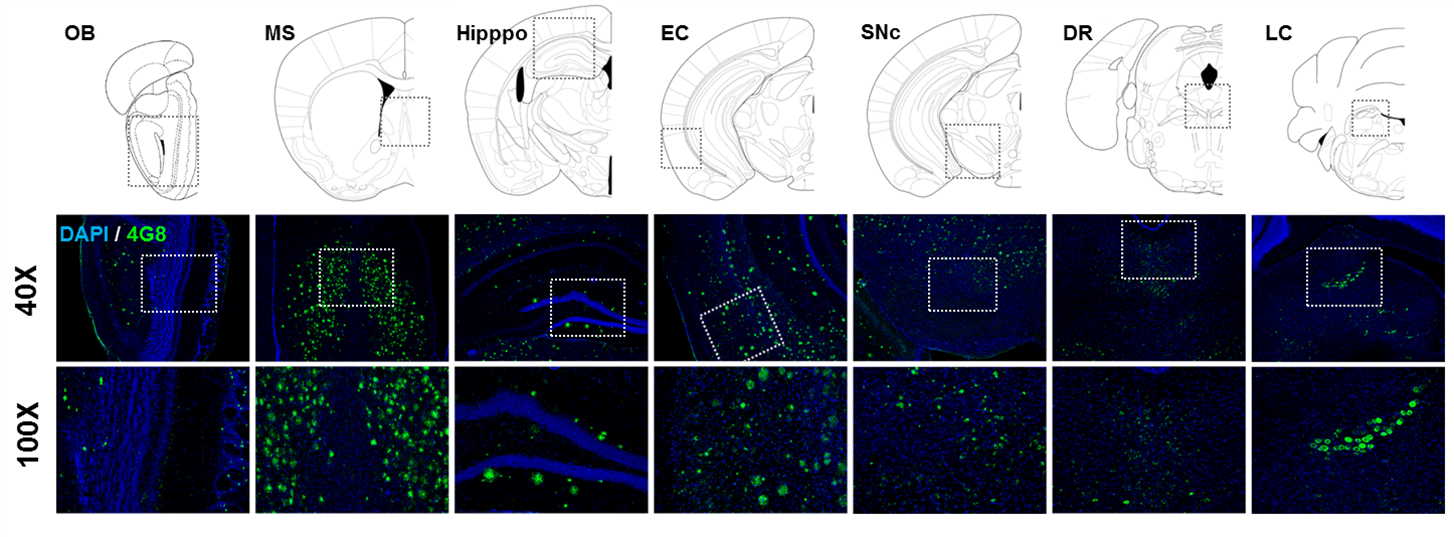

Supplement: Supplementary file 5 — Validation and profiling of the Fluoro-Gold-labeled afferent connections to the hippocampus. a Validation of the Fluoro-Gold injection site in the hippocampus. b Delineating the Fluoro-Gold-positive areas in the brain regions that project afferents to the hippocampus. Representative brain slices showing DiI-labeled cells. EC: entorhinal cortex, LC: locus coeruleus, MS: medial septum, DR: Dorsal raphe (311 KB) [file 12035_2018_918_Fig12_ESM.gif]

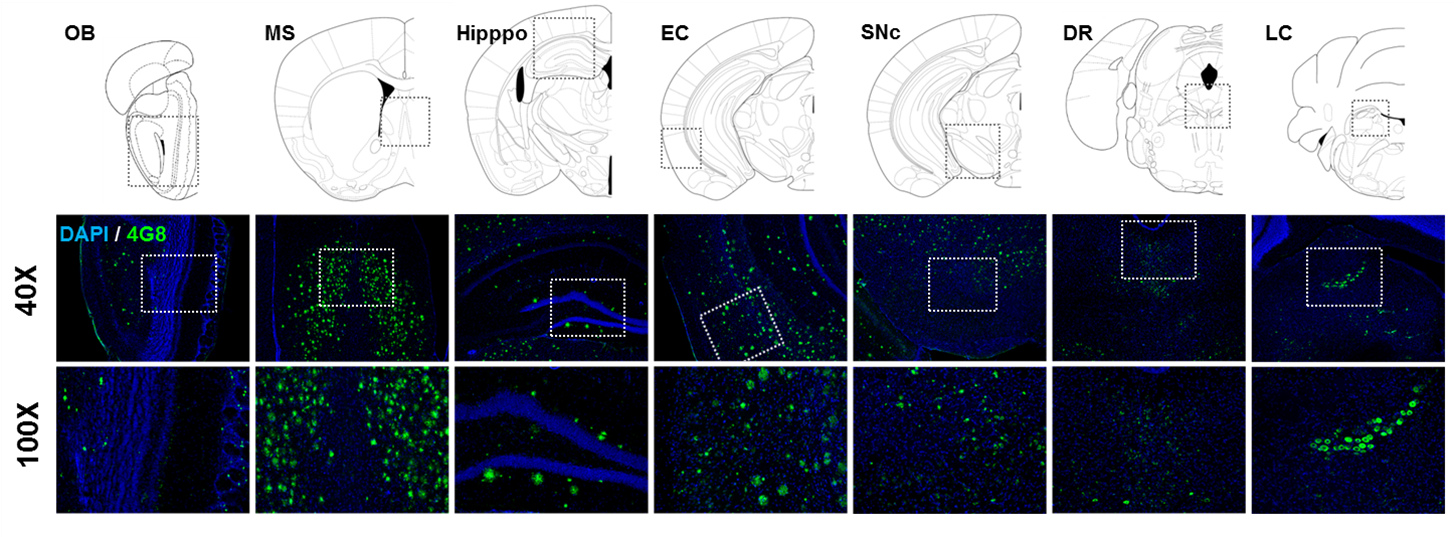

Supplement: Supplementary file 6 — (906 KB) [file 12035_2018_918_MOESM3_ESM.tif]

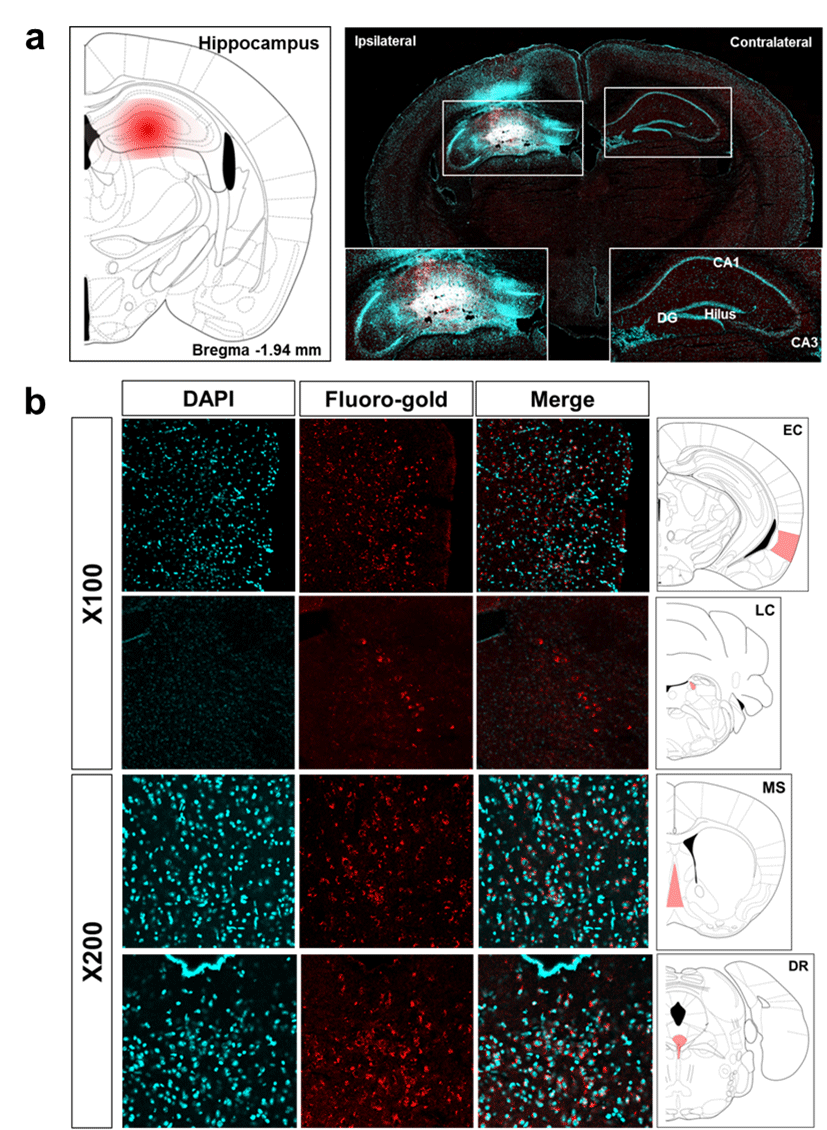

Supplement: Supplementary file 7 — Aβ-accumulation in the brains of the 5XFAD mice. The brain sections of the 5XFAD mice were stained with the 4G8 antibody to reveal Aβ plaque accumulation in the brain areas exhibiting the DiI-labeled cells (1.24 MB) [file 12035_2018_918_Fig13_ESM.gif]

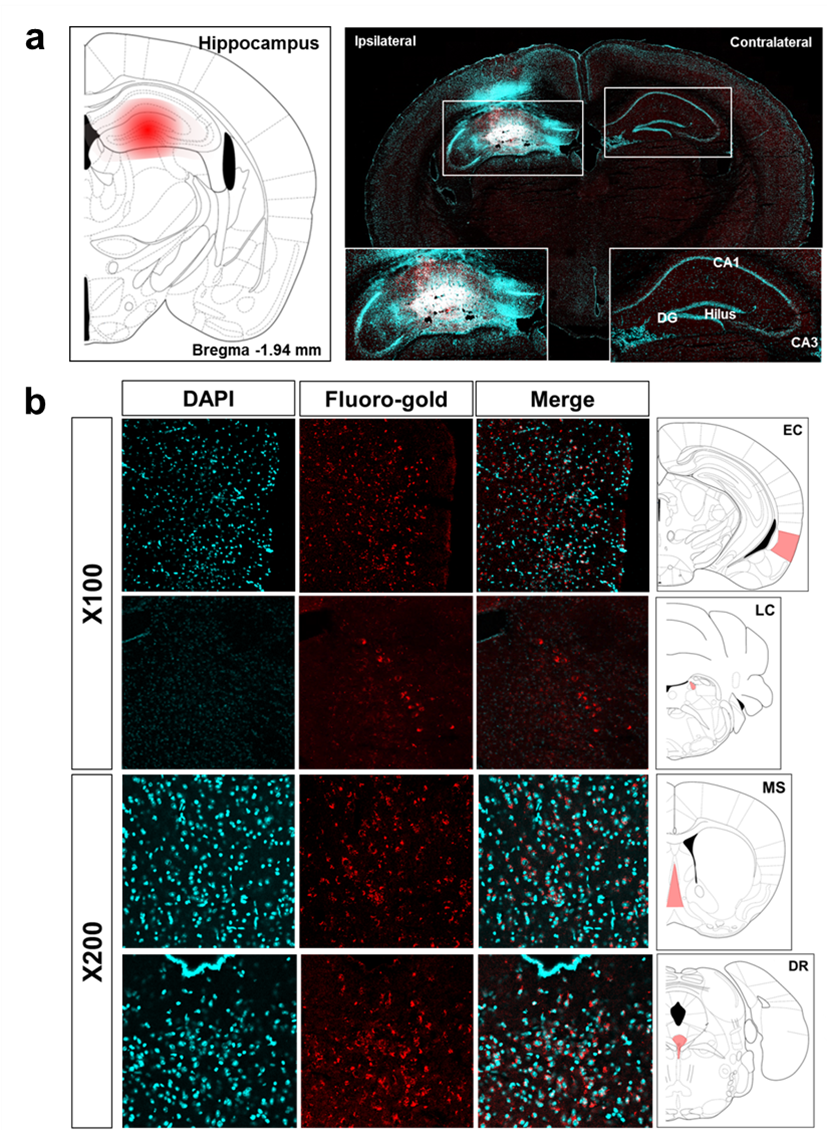

Supplement: Supplementary file 8 — (371 KB) [file 12035_2018_918_MOESM4_ESM.tif]
